# Spontaneous Rupture of the Inferior Cava

**Published:** 1845-01

**Authors:** 


*Spontaneous Rupture of the Inferior Cava.*—A young soldier, of a robust constitution, was seized on the 30th of December with pain in the abdomen, chiefly in the umbilical region; it was not increased on pressure, nor accompanied with any other marked symptom. The medical officer of the regiment regarded it as *enteralgic*, and prescribed rest and a gentle aperient. As the patient was no better in the evening, the cupping-glasses were applied on the right side of the abdomen, and gave immediate relief. He was left asleep at night, and early next morning was found dead in his bed. It deserves notice that this man had enjoyed excellent health up to the day when he was seized; the day before, he had mounted guard as usual.

*Dissection.*—The abdomen was much distended, and exhibited numerous dark patches of discolouration. On dividing its parietes, a large quantity of blood flowed out. It was long before we could find out whence it had proceeded. At length it was discovered that there was a rent—large enough to admit the point of the finger—in the inferior cava, on the level of the last dorsal vertebra: its edges were irregular and fringe-like. All the viscera were quite healthy.

It follows from the pathological appearances in this case discovered on dissection, 1st, that the death must have been rapid; and 2nd, that the perforation of the vena cava was probably consecutive upon an atrophy of the parietes of this blood-vessel.

The opinion of M. Vidal (de Cassis) on this subject is thus expressed in his "Traité de Pathologie Chirurgicale et de Medecine Operatoire":—"Atrophy, when extending to each of the membranes which compose a blood-vessel, or even affecting but one of them, necessarily weakens it, and sometimes reduces the vascular parietes to an extreme tenuity. Hence arises the fear of these ruptures, which have been observed in the superior cava, the venæ portæ, &c. These ruptures are either with or without some appreciable morbid lesion."

M. Andral, in his "Precis d'Anatomie Pathologique," cites a case in which, in the middle of a scuffle, a man in good health fell down suddenly, and expired in a few seconds. On opening the body, there was found a perforation of the abdominal vena cava. The edges of this perforation seemed as if it had been torn; but around it, the vessel appeared in a perfectly heathy state.—*Ibid, from Annales de la Chirurgie.*

